# Bioinformatics in Neonatal/Pediatric Medicine—A Literature Review

**DOI:** 10.3390/jpm14070767

**Published:** 2024-07-18

**Authors:** Dimitrios Rallis, Maria Baltogianni, Konstantina Kapetaniou, Chrysoula Kosmeri, Vasileios Giapros

**Affiliations:** 1 Neonatal Intensive Care Unit, School of Medicine, University of Ioannina, 45110 Ioannina, Greece; drallis@uoi.gr (D.R.); mbalt@doctors.org.uk (M.B.); 2 Department of Pediatrics, School of Medicine, University of Ioannina, 45110 Ioannina, Greece; k.kapetaniou@uoi.gr (K.K.); chrisa.kosmeri@gmail.com (C.K.)

**Keywords:** bioinformatics, database, genomics, neonate, website

## Abstract

Bioinformatics is a scientific field that uses computer technology to gather, store, analyze, and share biological data and information. DNA sequences of genes or entire genomes, protein amino acid sequences, nucleic acid, and protein–nucleic acid complex structures are examples of traditional bioinformatics data. Moreover, proteomics, the distribution of proteins in cells, interactomics, the patterns of interactions between proteins and nucleic acids, and metabolomics, the types and patterns of small-molecule transformations by the biochemical pathways in cells, are further data streams. Currently, the objectives of bioinformatics are integrative, focusing on how various data combinations might be utilized to comprehend organisms and diseases. Bioinformatic techniques have become popular as novel instruments for examining the fundamental mechanisms behind neonatal diseases. In the first few weeks of newborn life, these methods can be utilized in conjunction with clinical data to identify the most vulnerable neonates and to gain a better understanding of certain mortalities, including respiratory distress, bronchopulmonary dysplasia, sepsis, or inborn errors of metabolism. In the current study, we performed a literature review to summarize the current application of bioinformatics in neonatal medicine. Our aim was to provide evidence that could supply novel insights into the underlying mechanism of neonatal pathophysiology and could be used as an early diagnostic tool in neonatal care.

## 1. Introduction

Bioinformatics is a scientific field that uses computer technology to gather, store, analyze, and share biological data and information. It is a multidisciplinary field integrating biology, physics, mathematics, and computer science [1]. The types of data that bioinformatics use include DNA sequences of genes or entire genomes, protein amino acid sequences, nucleic acid, and protein–nucleic acid complex structures. Moreover, further data streams include proteomics, the distribution of proteins in cells, interactomics, the patterns of interactions between proteins and nucleic acids, and metabolomics, the types and patterns of small-molecule transformations by the biochemical pathways in cells. Currently, the objectives of bioinformatics are integrative, focusing on how various data combinations might be utilized to comprehend organisms and diseases. Due to the latest developments in the reading of DNA sequences, the difficulty in obtaining information has decreased, yet the comprehension and interpretation of the gathered data are still challenging. Considering the enormous size of the collected datasets, computer-based methods are currently the standard methods of interpretation and analysis.

Bioinformatic techniques have become popular as novel instruments for examining the fundamental mechanisms behind neonatal diseases. In the first few weeks of newborn life, these methods can be utilized in conjunction with clinical data to identify the most vulnerable neonates and to gain a better understanding of certain mortalities, including respiratory diseases, sepsis, or inborn errors of metabolism [2,3,4]. Reviews of the application of bioinformatics to neonatal care are scarce, and this review aims to cover this important issue. In the current study, we performed a narrative literature review to summarize the current application of bioinformatics in neonatal medicine. Our aim was to provide evidence that could supply novel insights into the underlying mechanism of neonatal pathophysiology and could be used as an early diagnostic tool in neonatal care.

**Figure 1:**
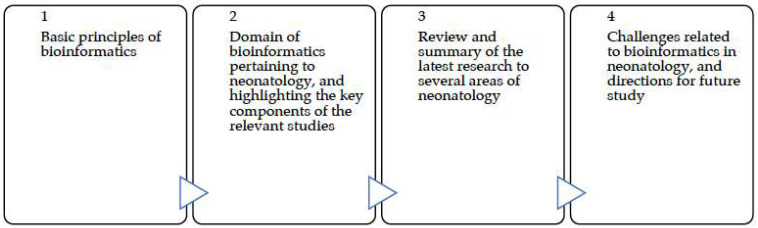
Overview of the study organization.

Our study is organized into (1) presenting the basic principles of bioinformatics, (2) grouping bioinformatics that pertain to neonatology into domains, elucidating their sub-domains, and highlighting the key components of the relevant studies, (3) reviewing and providing a thorough summary of the latest research to all areas of neonatology, and (4) examining and discussing the existing challenges related to bioinformatics in neonatology, as well as directions for future study (Figure 1).

## 2. Basic Principles of Bioinformatics

### 2.1. Bioinformatic Analysis 

Bioinformatics is primarily based on the internet and computer software, while a basic activity is the sequence analysis of proteins and DNA using different online databases and programs. Bioinformatics has expanded globally, establishing computer networks that facilitate the straightforward retrieval of biological data and the creation of software applications for analysis. Numerous global initiatives are underway to make gene and protein databases openly accessible online to the entire scientific community [1].

The growing volume of data resulting from genome research has made computer databases with quick assimilation, reusable formats, and algorithm software programs essential for effective biological data management [5]. Due to the diversity of new data, it is not possible to access all of this information in a single, complete database. Examples include websites that offer in-depth explanations of clinical conditions, a list of genetic mutations and polymorphisms associated with illness susceptibility, and the ability to search for disease genes based on a DNA sequence.

### 2.2. Bioinformatics Databases

To guarantee data transparency and traceability, several international collaborations and databases, or biorepositories, have been established [6]. These datasets are frequently combined to assist scientists in moving from identifying genetic alterations to figuring out which biochemical pathways the questioned genes are part of. These pathways assist in explaining the underlying physiology and give context to the findings. To name a few examples of databases and websites, the National Centre for Biotechnology Information (NCBI), the European Nucleotide Archive, the Gene Ontology (GO), the Ensembl, the Genome-Wide Association Study (GWAS) catalog, the Gene Expression Omnibus (GEO), the SWISS-PROT, and the Kyoto Encyclopedia of Genes and Genomes (KEGG) are frequently referenced in the literature (Table 1).

### 2.3. Functional Genomics

Functional genomics is the study and interpretation of biological data at the level of the transcriptome, proteome, and genome [7]. Clinical research and the molecular knowledge of diseases have made bioinformatics increasingly visible. Notably, this field covers several “omics” disciplines that enable a more thorough examination of biological systems, including proteomics (the study of proteins), metabolomics (the research of metabolites), transcriptomics (the study of transcripts), and genomics (the study of DNA), whereas more specialized disciplines, such as metagenomics, combine the study of the human genome with other organisms, such as bacteria, viruses, etc., and epigenomics studies epigenetic alterations of DNA [8]. For a more comprehensive examination, these distinct “omics” fields are frequently integrated and referred to as “multi-omics” or “panomics”.

Moreover, the DNA microarray technique incorporates genotyping and DNA sequencing and determines the degree of gene expression. In addition to analyzing genome sequence data, bioinformatics is currently being used for a wide range of other significant tasks, such as analyzing gene expression and variation, predicting and analyzing the structure and function of genes and proteins, identifying and predicting gene regulation networks, simulating whole-cell environments, modeling complex gene regulatory dynamics and networks, and presenting and analyzing molecular pathways to comprehend gene–disease interactions [9]. In bioinformatic protein research, a database of two-dimensional electrophoresis and annotated proteins is used, in order to predict the protein’s structure once it has been separated, identified, and characterized. Structural biologists also employ bioinformatics to manage the massive and complex data when creating three-dimensional models of molecules using electron microscopy, nuclear magnetic resonance, and X-ray crystallography [10]. Simpler bioinformatic activities, albeit on a lesser scale, that are useful to the clinical researcher can range from predicting the function of gene products to developing primers, which are short oligonucleotide sequences required for DNA amplification in polymerase chain reaction assays.

### 2.4. Translational Bioinformatics

The outcome of translational bioinformatics is “the transformation of increasingly voluminous biomedical data, and genomic data, into proactive, predictive, preventive, and participatory health”, according to the American Medical Informatics Association [11]. “Proact” refers to taking proactive measures to address a change or challenge that is anticipated. Even when there is not enough data to support a particular clinical choice, there is still another chance to use a proactive strategy to personalize care. “Predict” refers to stating or making known something before it is presented clinically, particularly by inference or specialized knowledge. Characteristics of the model itself may aid in improving the comprehension of the pathophysiology of a disease or in identifying hazardous behaviors that may be involved. “Prevent” describes actions that impede or lessen the progression of a disease. Prevention of prematurity is likely the best example of preventive treatment in the field of neonatology, even though there is some overlap in the care that is seen as proactive, predictive, and preventive. Finally, “participation” refers to when people, groups, and organizations are consulted about a project or program of activity or given the chance to actively participate in it [12].

## 3. Bioinformatics in Neonatal Medicine 

### 3.1. Methods

#### Literature Search Strategy 

**Figure 2:**
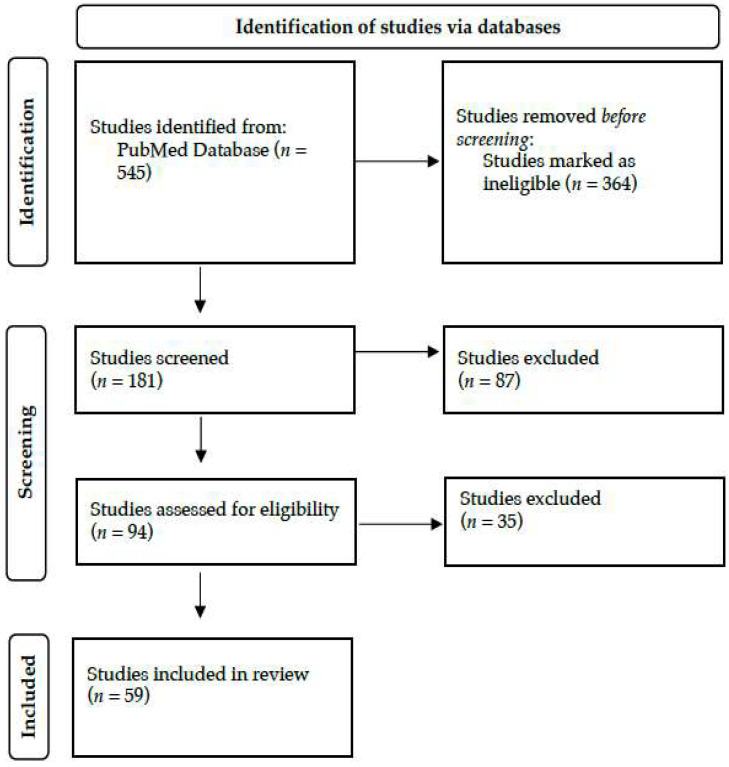
Literature search strategy and study selection, adopted by the PRISMA flow chart.

A literature search was conducted by two researchers in June 2024 in PubMed. Only human studies and English-language articles were taken into account. The terms ‘biomedical informatics’ OR ‘bioinformatics’ OR ‘computational biology’ OR ‘Kyoto Encyclopedia of Genes and Genomes’ OR ‘Genomes’ OR ‘Gene Ontology’ OR ‘Genome-wide association study’ OR ‘Biotechnology Information’ OR ‘gene dataset’ OR ‘SWISS-MODEL’ AND ‘neonate’ OR ‘newborn’ OR ‘infant’ OR ‘Neonatal Intensive Care Unit’ OR ‘Neonatology’ in the title or abstract were utilized. The studies that were retrieved were assessed according to their titles, abstracts, and suitability for the review. As outlined in Figure 2, 59 out of 545 studies were selected and included in this narrative review.

### 3.2. Results

#### 3.2.1. Applications of Bioinformatics in Neonatal Respiratory Diseases

- Respiratory distress syndrome

Neonatal respiratory distress syndrome (RDS) is very common among preterm neonates. Previously, Zhou et al., in 2021, explored the connection between circular RNA (circRNA) and the expression profile of circRNAs and RDS, performing high-throughput sequencing, and analysis with GO and KEGG [13]. The authors found 30 enriched KEGG pathways of 125 target genes engaged in the production and release of endocrine hormones associated with the development of RDS (Table 2) [13]. Overall, although additional molecular biology validation is required to precisely identify the function of differentially expressed circRNAs in neonatal RDS, circRNAs may serve as molecular markers for early RDS diagnosis, offering potential novel treatment options [13].

- Bronchopulmonary dysplasia

More extensively, bioinformatics analysis has been applied to the investigation of genetic variation associated with bronchopulmonary dysplasia (BPD) in preterm neonates (Table 2). By employing a DNA pooling technique on newborns with African and White ancestry, Hadchouel et al. discovered the *SPOCK2* gene as a novel, potentially susceptible gene for BPD [14]. Furthermore, Wang et al. used a GWAS in 2013 to analyze genomic DNA from newborn screening bloodspots and find genetic variations linked to the risk for BPD [15]. The authors analyzed samples from 1726 neonates, but they were not able to identify genomic loci or pathways that could explain BPD. The study’s results could be explained by genetic variants that were mapped to a large number of distributed loci, race and ethnicity, or the study population’s sample size [15]. In 2015, Ambalavanan et al. used a GWAS to locate single-nucleotide polymorphisms (SNPs) and pathways linked to BPD [16]. The authors discovered that the genes *miR-219* and *CD44* were upregulated in the lungs of BPD patients as well as in relation to hyperoxia. A comparison of the pathways linked to mild/moderate and severe BPD revealed variations in these pathways, suggesting that these novel components and pathways may be involved in lung development and repair and genetic susceptibility to BPD [16]. Moreover, Mahlman et al., in 2017, performed a GWAS on preterm neonates (24–30 weeks of gestational age) and revealed that SNPs close to the C-reactive protein (CRP) gene were risk factors for BPD, independent of antenatal risk factors [17]. Therefore, the authors proposed a potential role for variants near CRP in BPD [17]. Yang et al. assessed the expression patterns of matrix metalloproteinase (MMP) and angiogenesis-related genes (ARG) in neonates with and without BPD. Using the Gene-Cloud of the Biotechnology Information platform, the authors re-analyzed the GEO database dataset [18]. The study found that by interfering with the development of blood vessels, the up- and down-regulation of particular genes may increase the risk of BPD in preterm neonates [18]. Furthermore, Torgerson et al., in 2018, used ancestry studies and a GWAS to detect genes, pathways, and variants linked to survival in BPD neonates receiving inhaled nitric oxide [19]. Pathway analyses revealed variation in genes involved in immune/inflammatory processes in response to infection and mechanical ventilation, and examination of the genes upregulated in BPD lungs revealed an association with variants in a cytokine linked to fibrosis and interstitial lung disease [19]. Overall, the study indicated that genetic variations in immune response, drug metabolism, and lung development influence individual and racial/ethnic variations in respiratory outcomes when high-risk preterm neonates receive inhaled nitric oxide. Finally, using GO enrichment and KEGG pathway analysis, Wang et al. conducted a study to investigate differentially expressed exosomal circRNAs, long noncoding RNAs (lncRNAs), and messenger RNAs (mRNAs) in the umbilical cord blood of newborns with and without BPD [20]. The study’s conclusions demonstrated a substantial difference in expression in the exosomes obtained from umbilical cord blood between newborns with and without BPD, underscoring the possible biological roles of exosomal circRNAs and lncRNAs in BPD [20]. 

Nonetheless, whole-exome sequencing (WES) made it possible to investigate uncommon variations associated with BPD. In 2015, Carrera et al. identified potential candidate genes linked to the development of BPD by using WES in 26 unrelated newborns with severe BPD [21]. Among 3369 new variants found, the toll-like receptor family, *NOS2, MMP1, CRP, LBP*, and other top candidate genes were identified [21]. Furthermore, in 2022, Wang et al. performed an epigenome-wide association analysis in preterm neonates, utilizing cord blood DNA and DNA methylation techniques, providing insights into the molecular mechanisms involved in BPD etiology [22]. The study revealed that the incidence of stochastic epigenetic mutations at birth was considerably higher in patients with BPD, while changes in the transcriptome of cord blood cells were indicative of BPD disease [22]. In conclusion, the authors suggested that DNA methylation profiles in preterm cord blood were significantly altered by the nucleated red blood cell concentration, and epigenome-wide association study analysis provided possible insights into the molecular processes implicated in the pathophysiology of BPD [22].

In line with the development of genomics, proteomic analysis has also been used for identifying specific protein–BPD associations. Magagnotti et al., in 2013, discovered distinct variations in the proteomic profiles of preterm neonates born between 23–25 and 26–29 weeks of gestational age, as well as between neonates diagnosed with mild and severe BPD, utilizing proteome analysis in tracheal aspirates [23]. Also, Ahmed et al. performed proteomic analysis on the urine of neonates with BPD in a study that was published in 2022 [24]. They validated several proteins previously discovered in serum samples and tracheal aspirates that had been linked to the pathogenesis of BPD, providing a means of non-invasively tracking the disease’s progression over time [24]. The findings of the above studies could be used to help create new, successful treatments and therapeutic interventions for neonates with BPD in future studies.

- Cystic fibrosis

In 2024, Esposito et al. investigated whether newborn screening programs could aid in early identification and enhance the prognosis of neonates suffering from cystic fibrosis (Table 3) [25]. With the use of Sanger-sequencing-based molecular techniques and bioinformatics tools, the scientists were able to identify an Alu element insertion in exon 15 of the cystic fibrosis transmembrane conductance regulator (CFTR) gene, which has a significant impact on splicing patterns, CFTR protein structure, and gene expression. In summary, the study underscored the significance of how the combination of contemporary technologies and human skills signified a crucial advancement in the field of genetic medicine [25].

#### 3.2.2. Applications of Bioinformatics in Cardiovascular Disorders

Bioinformatics analysis has also been applied to the investigation of genetic variation and significant differences in genome-wide DNA methylation in neonates with congenital heart defects (CHD), as depicted in Table 4. When Bahado-Singh et al. analyzed the genome-wide DNA methylation of neonates with different CHDs, such as hypoplastic left heart syndrome, ventricular septal defect, atrial septal defect, pulmonary stenosis, coarctation of the aorta, and Tetralogy of Fallot, they discovered significant variations in the cytosine methylation of hundreds of genes [26]. In 2020, the same group investigated whether isolated, non-syndromic coarctation of the aorta results in notable epigenetic alterations. Six artificial intelligence platforms, including deep learning, and biological and disease pathways that were epigenetically dysregulated were identified by the scientists using ingenious pathway analysis [27]. According to the study, the newborn blood spot might be used to accurately predict the coarctation of the aorta, and artificial intelligence and epigenomics could be utilized to accomplish key goals of precision cardiovascular therapy [27]. Similarly, to investigate the epigenetic alterations that occur in neonates with Tetralogy of Fallot, Radhakrishna et al. conducted a genome-wide methylation analysis [28]. Significant biological processes and functions associated with differentially methylated genes were found by GO analysis, which provided important insights into the pathophysiology of Tetralogy of Fallot [28]. Furthermore, Rashkin et al. utilized transmission/disequilibrium tests in complete case-parental trios and case-control analyses separately in infants and mothers to investigate the genetic architecture of obstructive heart diseases, and found an association between two specific SNPs and obstructive heart diseases [29]. In line with the previous authors, Mouat et al. [30], Huang et al. [31], and Wang et al. [32] investigated genetic variance and methylation differences in neonates with CHDs, indicating that bioinformatics may be useful for future efforts to improve genetic screening and patient counseling.

#### 3.2.3. Applications of Bioinformatics in Neonatal Gastrointestinal Disorders

Several studies also explored the relation of human milk components with the development of gut microbiota (Table 5). Wang et al. recently investigated the expression of lactation-related miRNAs in microvesicles isolated from the umbilical cord blood, with Western blotting, transmission electron microscopy, and nanoparticle tracking analysis, while bioinformatics techniques for GO, miRNA target prediction, signaling pathway analysis, and lactation-related miRNAs were performed [33]. After profiling 337 miRNAs in human umbilical cord blood microvesicles, bioinformatics analysis revealed that 85 of them were connected to lactation [33]. According to the authors, umbilical cord blood microvesicles may play a significant role in fetal–maternal interaction by mediating β-casein secretion through miRNAs [33]. Similarly, Parnanen et al., in 2022, determined the impact of early exposure to formula on the antibiotic-resistance genes’ load, using a generalized linear model that was constructed using cross-sectionally sampled neonatal gut metagenomes to examine the effect of food on antibiotic-resistance genes’ loading in neonates, while neonatal metagenomes collected from public databases were used to cross-validate the model [34]. The study revealed that, when compared to neonates that were exclusively fed human milk, the formula-receiving group had a 69% greater relative abundance of antibiotic-resistance genes carried by gut bacteria [34]. Liu et al. more recently investigated the characteristics of gut microbiota dysbiosis and metabolite levels in very or extremely low-birth-weight neonates with white matter injury (WMI) by LC-MS/MS, diffusion tension imaging, and 16S rRNA gene sequencing as part of a multi-omics approach, and they found that there was a significant differential expression of 139 metabolic markers between WMI and non-WMI neonates [35]. Finally, Letourneau et al., in 2024, investigated the association between microbiome composition and biomarkers and the risk of developing specific diseases, and showed that neonates born earlier or exposed to antibiotics exhibited increased fecal pH and increased redox, while microbiome composition was also related to birth weight, gestational age, pH, and redox [36]. 

Apart from the research on the gut microbiota, a recent study evaluated the role of bioinformatics analysis in detecting neonates at risk of necrotizing enterocolitis (NEC) (Table 5). Liu et al. explored the differentially expressed genes in neonates with NEC, using the GEO database, GEO2R, DAVID, and STRING to examine the roles, pathway enrichment, and protein interactions of the associated genes, while Cytoscape software (https://cytoscape.org/) was used to identify the important protein interaction modules and core network genes [37]. The findings showed that the differentially expressed genes that were upregulated were associated with protein dimerization activity, whereas the differentially expressed genes that were downregulated were associated with cholesterol transporter activity, suggesting that biological mechanisms and metabolic pathways might be crucial in the development of NEC [37]. Chen et al., in 2021, investigated the effects of human-milk-derived exosomes in the gut microbiota, revealing that the function of intestinal epithelial cells is regulated by the top 50 lipids through the extracellular signal-regulated kinase/mitogen-activated protein kinase (ERK/MAPK) pathway [38]. The findings of the study revealed the lipidomic complexity in exosomes obtained from term and preterm milk and offered a new mechanistic understanding of how human milk inhibits the development of NEC [38]. Furthermore, Zhang et al. investigated biological function, pathways, transcription factors, and immune cells dysregulated in NEC using gene set enrichment analysis and found that both innate and adaptive immune systems may trigger the NEC-related inflammatory response [39]. Lastly, to examine biological and functional processes that may be involved in the pathophysiology of NEC, Tremblay et al. employed functional enrichments with the GO and the KEGG databases to evaluate earlier data [40]. The authors found that the most significant biological pathways that were over-represented in neonates with NEC were strongly related to innate immune systems. The study thus suggested that more research is necessary to precisely understand the function of inflammatory genes connected to the IL-17 pathway and its downstream targets in NEC [40]. 

Niu et al. used the Database for Annotation, Visualization, and Integrated Discovery to perform GO and KEGG pathway enrichment studies on differently expressed genes (DEGs) from public datasets in order to discover key genes involved in the development of Hirschsprung’s disease [41]. Weighted gene co-expression network analysis was used to create the co-expression network between lncRNAs and mRNAs. The authors proposed that hub mRNAs and hub lncRNAs may be involved in the development of Hirschsprung’s disease and that these genes may offer novel clinical indicators for assessing the disease’s risk [41]. Besides, Feng et al. explored the underlying mechanism of enteric neural precursor cells (ENPCs) and the ZEB2/Notch-1/Jagged-2 pathway in Hirschsprung’s-associated enterocolitis development by Western blot and RT-qPCR, while bioinformatics analysis and co-immunoprecipitation were utilized to investigate the ZEB2 and Notch-1 interaction [42]. It was found that Hirschsprung’s-associated enterocolitis colon tissues had higher levels of lipopolysaccharide, along with downregulated ZEB2 and elevated Notch-1/Jagged-2 expression. In lipopolysaccharide-induced ENPCs, overexpression of ZEB2 exacerbated inflammation and dysfunction while suppressing Notch-1/Jagged-2 signaling, thus playing a role in Hirschsprung’s-associated enterocolitis [42]. 

#### 3.2.4. Applications of Bioinformatics in Neonatal Sepsis

Gene polymorphisms, biomarkers, and metabolomics have also been investigated in neonates with sepsis (Table 6). Mustarim et al. evaluated the association between several gene polymorphisms and the incidence of neonatal sepsis by PCR examination, sequencing, and bioinformatics analysis [43]. The authors found a significant correlation between the *Interleukin 1β* rs1143643 G>A gene polymorphism and the frequency of newborn sepsis [43]. Bu et al., evaluating upregulated and downregulated mRNAs and lncRNAs in neonatal sepsis, by constructing protein–protein interaction networks, demonstrated that neonatal sepsis was associated with 1128 upregulated and 1008 downregulated mRNAs, and 28 upregulated and 61 downregulated lncRNAs [44]. Thus, the findings could help detect new therapeutic markers for neonatal sepsis [44]. Navarrete et al. utilized two distinct bioinformatic approaches (a supervised and an unsupervised) using data from methylation arrays of leukocytes, and they managed to identify variation in DNA methylation traits in neonatal sepsis, as well as between neonates with early compared to late-onset sepsis [45]. Yan et al. tried to identify the optimal biomarkers in the progression of neonatal sepsis by gene set variation analysis (GSVA), CIBERSORT, receiver operating characteristic analysis, and the LASSO model [46]. The authors found that according to the GSVA data, differentially expressed genes mostly influenced the upregulation of metabolism-related activities and inflammation, as well as the suppression of adaptive immune responses in sepsis. Ultimately, three genes were shown to be important biomarkers for sepsis, providing novel insights into the pathogenesis and promising therapeutic options of neonatal sepsis [46]. Additionally, Ciesielski et al. conducted an exploratory GWAS to find genetic variations linked to late-onset sepsis and concluded that NOTCH signaling was over-represented based on pathway studies [47]. 

Furthermore, Liu et al. investigated the characteristics of intestinal metabolomics and non-invasive biomarkers for late-onset sepsis by analyzing gut metabolites in preterm neonates and suggested that several metabolites (N-methyldopamine, cellulose, glycine, N-ribosylnicotinamide, Gamma-glutamyltryptophan, and 1-alpha, 25-dihydroxycholecalciferol) demonstrated distinct diagnostic values as non-invasive biomarkers for late-onset sepsis [48]. Also, Das et al., in 2024, examined the blood profile of very preterm neonates across episodes of sepsis with multi-parameter flow cytometry, single-cell RNA sequencing, and plasma analysis, and they found that a blood immune signature was present even in cases where CRP was normal. Single-cell RNA sequencing revealed elevation of amphiregulin in leukocyte populations during sepsis, which was associated with clinical indications of disease [49]. Furthermore, utilizing the GEO public repository to extract programmed cell death (PCD)-related genes from 12 distinct patterns and sophisticated machine learning methods, such as LASSO, support vector machine-recursive feature elimination (SVM-RFE), protein–protein interaction (PPI) networks, artificial neural networks, and consensus clustering, Hang et al. investigated whether PCD could function as a marker for diagnosing neonatal sepsis [50]. According to the study, the competing endogenous RNA (ceRNA) network showed a complex regulatory interplay based on the identified marker genes, and the immune infiltration analysis indicated considerable discrepancies in neonates diagnosed with sepsis [50]. Lastly, Zhao et al. examined the expression patterns of particular miRNAs and assessed their diagnostic utility for the early identification and management of sepsis by GO enrichment and PPI studies [51]. The three miRNA panels (miR-15a-5p, miR-223-3p, and miR-16-5p) may provide a unique non-invasive biological marker for EOS screening, according to the study’s overall findings [51].

#### 3.2.5. Applications of Bioinformatics in Neonatal Neurology 

Recent advances in neonatal care have changed the management and prognosis of neonates with hypoxic-ischemic encephalopathy (HIE); therefore, interest has been focused on detecting prognostic patterns for suffering neonates (Table 7). Since 2006, Chu et al. investigated the metabolomic patterns of newborn urine samples with clinical indications of severe hypoxia at birth, using bioinformatic techniques, including hierarchical clustering analysis [52]. The authors found that inhibited biochemical networks involved in macromolecular production were associated with HIE, as elevated levels of eight urine organic acids in different biochemical pathways were found to be highly sensitive and specific indicators of the prognosis of neurodevelopmental impairment [52]. Moreover, Zhu et al. identified potential biomarkers of neonatal HIE, via the isobaric tags for absolute and relative quantification (iTRAQ) method, and bioinformatics investigations, such as GO and KEGG pathway enrichment analysis [53]. The authors found 51 frequently differently expressed proteins in neonates with HIE compared to controls [53], indicating haptoglobin and S100A8 as potential biomarkers for neonatal HIE, also reflecting the severity of the disease [53]. Furthermore, to investigate the processes of injury and recovery in neonatal encephalopathy, Friedes et al. used liquid chromatography with tandem mass spectrometry (LC/MS/MS) to undertake a targeted metabolomic study [54]. The two-year neurodevelopmental outcomes, as assessed by the Bayley Scales of Infant and Toddler Development III, were compared to metabolite levels. The authors proposed that plasma metabolites could improve existing clinical predictors and aid in the prediction of neurological outcomes in infant brain damage using KEGG pathways [54].

Furthermore, several studies have been focused on evaluating the diagnostic performance of the newborn screening program (NBS) on inborn errors of metabolism (IEMs). Tangeraas et al. evaluated the performance of the Norwegian expanded NBS, including a total of 21 IEMs, and they found that the incidence of IEMs increased by 46%, mostly as a result of the discovery of attenuated phenotypes, after the expanded NBS was implemented [3]. Also, Hagemeijer et al. focused on the improvement of the detection of lysosomal storage disorders (LSDs), utilizing ultra-high-performance liquid chromatography/high-resolution accurate mass (UHPLC/HRAM) mass spectrometry screening technology, combined with an open-source iterative bioinformatics process [55]. The authors demonstrated that several LSDs were associated with abnormal urine oligosaccharide excretions, which could be potential urine biomarkers for the latter diseases [55]. Moreover, Sabi et al. examined through the NBS program, additional biomarkers for distinguishing falsely suspected glutaric aciduria type-1, by utilizing liquid chromatography–high-resolution mass spectrometry (LC-HRMS), and they revealed several up- and down-regulated metabolites in transient disease [56]. Thus, the findings of the study suggested that a unique metabolic pattern associated with the transient rise in metabolites improves the prediction of falsely positive cases, potentially reducing the need for needless medical interventions [56].

Finally, Chung et al., in 2024, examined four prediction models for cognitive or motor function at 24 months of corrected age, using hospitalized and follow-up data of very preterm neonates that were analyzed using an evolutionary-derived machine learning technique, called EL-NDI, and compared to each other using random forest, SVM, and LASSO regression [57]. The EL-NDI model, using ten variables for cognitive delay and four variables for motor delay, respectively, achieved comparable predictive performance to other models using 29 or more variables [57]. 

Of note, several case-report studies have been published reporting bioinformatic applications for detecting specific IEMs (Table 8). Maryami et al. reported the use of whole-exome sequencing (WES) analysis in combination with different approaches of bioinformatics analysis for detecting metabolic crises on the background of IEMs in the early neonatal period [58,59,60]. Similarly, Forte et al. [61] and Wei et al. [62] reported the use of WES analysis, Sanger analysis, and bioinformatic application for the detection of the pathogenic variants of the galactose-1-phosphate uridylyltransferase gene and the polycystic kidney disease-1 gene, respectively. 

#### 3.2.6. Miscellaneous Applications of Bioinformatics in Neonatal Medicine 

A study by Lu et al. in 2006 investigated PAX3 SNPs that may be linked to syndromic neural tube abnormalities (Table 9). The results showed that certain variants of the PAX3 gene were linked to a higher incidence of spina bifida among Hispanic White neonates. [63]. Furthermore, Pan et al. investigated the expression of new noncoding RNAs, called circRNAs, between neonates with hypoxia-induced acute kidney injury and controls, using high-throughput sequencing [64]. The authors demonstrated that 112 circRNAs were considerably downregulated in the acute kidney injury group, while 184 were noticeably elevated and, thus, the findings could contribute to future research on neonatal acute kidney injury and facilitate the detection of novel therapeutic targets [64]. In a different aspect, Shipton et al. investigated the practicability of collecting and analyzing tear proteins from preterm infants at risk of retinopathy of prematurity (ROP), which might be implicated in the pathophysiology and prognosis of ROP, using MS for proteomic analysis [65]. The findings of the study suggested that an increase in the lactate dehydrogenase B chain in tears was associated with an increased risk of ROP [65]. Nonetheless, Marom et al. aimed to assess the rapid trio genome sequencing clinical value, diagnostic effectiveness, and viability in all of Israel’s neonatal intensive care units, via sequencing analysis and questionnaires to evaluate clinical utility [66]. The authors revealed a 50% diagnostic effectiveness for disease-causing variations, 11% for variants of unknown significance suspected of being the cause, and 1% for one unique gene candidate [66]. Finally, by analyzing whole-genome sequencing and clinical data using genotype-first and phenotype-first approaches, Pavey et al. assessed the potential of genomic sequencing to supplement the current newborn screening for immunodeficiency. Their findings suggested that neonatal genomic sequencing could potentially supplement newborn screening for immunodeficiency [67].

Finally, in case-report studies (Table 10), in two newborns with congenital central hypothyroidism with anemia resistant to conventional treatment, Baquedano et al. revealed the molecular effects of a unique missense mutation and a novel splice-junction mutation in the thyroid-stimulating hormone (TSH)-beta subunit gene [68]. Moreover, Zheng et al. explored the utility of WES to establish the diagnosis of congenital dyserythropoietic anemia type II, revealing that the analysis by multiple bioinformatics tools predicted that the mutant proteins were deleterious [69]. Besides, Khabou et al., in 2024, reported that they managed to establish the diagnosis of progressive familial intrahepatic cholestasis in six unrelated Tunisian infants, via panel-target sequencing, followed by an exhaustive bioinformatics and modeling investigations [70]. 

## 4. Challenges and Ethical Issues of Bioinformatics

Bioinformatics has aided in establishing networks that facilitate the retrieval of biological data, providing new insight into many complex neonatal diseases. The comprehensive interpretation of gene variation and molecular pathways that are involved in the pathogenesis of several neonatal diseases, such as RDS, BPD, CHD, NEC, HIE, and IEMs, could provide several promising therapeutic options. However, though the amount of information being generated increases daily, it is challenging to establish the optimum time and ways to incorporate it. Neonatal diseases are complex and multifactorial and thus, the concept that “one SNP causes one phenotype” is unsatisfactory. Further research is warranted to explore the complex gene–gene and gene–environment interactions [71]. Furthermore, although GWAS has been widely used to establish the relationship between SNPs and diseases, the biological link between genetic variations and phenotypic features is rarely disclosed [72]. Therefore, a systems biology-based strategy combining data from several biological levels, including the genome, transcriptome, and proteome, may be beneficial in understanding these links [73].

Nonetheless, bioinformatics research incorporated with artificial intelligence algorithms must adhere to ethical and impartial standards [74]. Confidentiality and privacy of sensitive patient data must be protected [75], whereas potential medico-legal risks and issues with insurability if unfavorable long-term results are anticipated should be addressed [76]. Future studies must strike a balance between the increased uncertainty and anxiety that parents and carers may experience as a result of these discoveries and the ethical implications of beneficence.

## 5. Future Directions 

Due to the recent advance in bioinformatics, the study of genetic disorders is shifting beyond the isolation of single genes and toward the discovery of gene networks within cells, the comprehension of intricate gene interactions, and the determination of the function of these networks in neonatal diseases [77]. Clinicians and clinical researchers will benefit from bioinformatics’ guidance and assistance in leveraging computational biology’s benefits [78]. Nonetheless, the clinical research teams who can transition with ease between the laboratory bench, neonatal clinical practice, and the use of these advanced computational tools will benefit the most in the upcoming decades. In addition, the role of artificial intelligence in the modern era has become an important partner in healthcare services. The main advantage of artificial intelligence is that provides clinicians the ability to evaluate large volumes of medical data that are too complex for medical professionals to study quickly enough to find the diagnosis and determine a treatment plan. After proper training, artificial intelligence models can function similarly to human neurons and support decision-making algorithms. Thus, in the following decades, clinicians could benefit from the advantages of using large bioinformatics datasets evaluated with artificial-intelligence-based models.

## 6. Conclusions

Bioinformatics is becoming popular as a novel instrument for examining the fundamental mechanisms behind neonatal diseases. Several studies have explored the gene expression and molecular pathways in neonatal RDS, BPD, CHDs, gut microbiota, NEC, sepsis, or IEMs. Further studies are, however, warranted to investigate complex gene–gene and gene–environment interactions in light of the variability of many neonatal disease symptoms and the multifactorial nature of their origin. 

## Figures and Tables

**Table 1:** Examples of bioinformatic databases and websites.

Database	Website (URL accessed on 12 July 2024)	Function
Nucleotide Sequence Databases		
Nucleotide	https://www.ncbi.nlm.nih.gov/nuccore	Database including sequences from the PDB, GenBank, RefSeq, TPA, and other sources.
GenBank	www.ncbi.nlm.nih.gov/Genbank	Repositories and records of DNA sequences from both individual labs and large-scale genome studies.
European Nucleotide Archive	https://www.ebi.ac.uk/ena/browser/home	European Nucleotide Archive: an extensive global database of nucleotide sequencing data.
Protein Sequence Databases		
Protein	https://www.ncbi.nlm.nih.gov/protein	A database including sequences from multiple sources, such as records from SwissProt, PIR, PRF, and PDB, and translations from annotated coding sections in GenBank, RefSeq, and TPA.
Protein Information Resource	https://proteininformationresource.org/pirwww/	An integrated public bioinformatics resource to help in systems biology, proteomics, and genomics research, called Protein Information Resource.
Gene Databases		
Gene	https://www.ncbi.nlm.nih.gov/gene	Integrates information from a wide range of species.
GeneCards	https://www.genecards.org/	An integrated, searchable database offering thorough, comprehensible details on every annotated and predicted human gene. Gene-centric data, including genomic, transcriptomic, proteomic, genetic, clinical, and functional information, are automatically included in the knowledgebase from over 150 web sources.
Gene Ontology	http://geneontology.org/	Established a link between research on humans and animals and the biological function of a gene.
Genome Databases		
Ensembl	https://www.ensembl.org/index.html	Ensembl is a genome browser that facilitates studies on transcriptional control, evolution, comparative genomics, and sequence variation for vertebrate genomes.
UCSC Genome Browser	https://genome.ucsc.edu/	Combines working model assemblies and reference sequences for a sizable genome collection at the University of California, Santa Cruz.
Genome Data Viewer	https://www.ncbi.nlm.nih.gov/gdv/	A genome browser designed for the examination and investigation of eukaryotic RefSeq genome assemblies.
Genome	https://www.ncbi.nlm.nih.gov/genome	Integrates genomic data, such as sequences, maps, chromosomes, assemblies, and annotations.
Genomes Online Database	https://gold.jgi.doe.gov/	A comprehensive site on the Internet that provides information about genome and metagenome sequencing initiatives and the metadata related to them.
Genome Analysis		
GeneCensus	http://bioinfo.mbb.yale.edu/genome/	Comparisons of genomes with respect to protein family sharing and metabolic pathway activity.
Genome-Wide Association Study (GWAS) catalog	https://www.ebi.ac.uk/gwas/	The human Genome-Wide Association Studies catalog maintained by the NHGRI-EBI.
National Center for Genome Resources	www.ncgr.org/	Creates software, collaborates, and provides information to link biologists with bioinformatics resources.
Gene Expression Databases		
Gene Expression Omnibus	https://www.ncbi.nlm.nih.gov/geo/	Individual gene expression profiles from carefully chosen datasets are available in the Gene Expression Omnibus repository.
Expression Atlas	https://www.ebi.ac.uk/gxa/home	Provides information regarding the expression of genes and proteins.
Gene Regulation Databases		
miRBase	https://www.mirbase.org/	Searchable published miRNA sequences and annotations can be found in the microRNA database.
The Encyclopedia of DNA Elements (ENCODE)	https://www.genome.gov/Funded-Programs-Projects/ENCODE-Project-ENCyclopedia-Of-DNA-Elements#al-5	To discover every functional element in the human and mouse genomes, a public research partnership was formed.
Protein Domain Databases		
InterPro	https://www.ebi.ac.uk/interpro/	A source offering protein sequence functional analysis.
The Human Protein Atlas	https://www.proteinatlas.org/	With the use of immunohistochemistry, the human protein atlas illustrates the expression and location of proteins in a wide range of healthy human tissues, cancer cells, and cell lines.
SWISS-PROT	www.expasy.org/sprot/	Large protein database with minimally redundant (few duplicate copies) sequencing data (including function, structure, and variations).
Protein Interaction Databases		
STRING	https://string-db.org/	A web server for protein–protein interactions.
BioGRID	https://thebiogrid.org/	Protein, genetic, and chemical interaction database.
Reactome	https://reactome.org/	A pathway database that is peer-reviewed, carefully curated, and available to the public.
Pathway Databases		
Kyoto Encyclopedia of Genes and Genomes	https://www.genome.jp/kegg/	Connects the information from genome sequencing to the appropriate cellular or biological processes.
Pathguide	http://www.pathguide.org/	An overview of more than 190 web-accessible biological route and network databases is provided by this meta-database.

**Table 2:** Original studies in neonatal respiratory diseases.

Reference	Aim	Method	Population	Outcome
Neonatal Respiratory Distress Syndrome				
Zhou et al., 2021 [13]	To explore the profile of expression and the association between circRNA and RDS.	CircRNA was analyzed with high-throughput sequencing, and the corresponding genes with GO and KEGG. Correlation between miRNA and its target genes was predicted using bioinformatics techniques.	10 neonatal samples were analyzed in microarray and 10 samples were used for real-time qPCR experiment.	30 enriched KEGG pathways of 125 target genes associated with the development of RDS. CircRNAs may serve as molecular markers for early RDS diagnosis, offering potential novel treatment options.
Bronchopulmonary Dysplasia				
Hadchouel et al., 2011 [14]	To find genetic variations linked to BPD.	Neonates of African and White heritage underwent a DNA pooling technique utilizing GWAS. The polymorphisms linked to BPD were verified in a separate replication population.	418 preterm neonates and 215 Finish neonates as a replication population.	Both series identified the *SPOCK2* gene. Individual genotyping and the replication population supported the presence of the most important variation (rs1245560). The correlation between rs1245560 and BPD in both African and White populations was validated by fine mapping. Also, rs1049269 was associated with BPD in White neonates.
Wang et al., 2013 [15]	To determine which genetic variations are associated with the risk of BPD.	For the GWAS, genomic DNA from newborn screening bloodspots from case and control groups was used. SNPs found in the HumanExome BeadChip and in the discovery GWAS were examined in the replication analysis.	1726 neonates.	No SNPs associated with BPD were found by genome-wide significance genotyping.
Ambalavanan et al., 2015 [16]	To identify SNPs and pathways associated with BPD.	GWAS was conducted on 1.2 million genotyped SNPs, and an additional 7 million imputed SNPs, using a DNA repository of extremely low-birth-weight neonates.	751 neonates.	While several SNPs in *CD44*, adenosine deaminase, and other genes were slightly below significance, no SNPs reached GWAS significance. Out of over 8000 pathways, 95 for severe BPD/death, 90 for severe BPD in survivors, and 75 for BPD/death were significant. The miR-219 targets for BPD/death and phosphorous oxygen lyase activity for both severe BPD/death and severe BPD in survivors were the pathways with the lowest false discovery rate. Analysis of gene expression revealed markedly elevated levels of *CD44* and *miR-219* in BPD, suggesting that these novel components and pathways may be involved in lung development and repair and the genetic susceptibility to BPD.
Mahlman et al., 2017 [17]	To identify gene loci predisposing neonates to BPD.	The initial GWAS included Finnish preterm neonates. The most promising SNPs associated with BPD were genotyped in both Finnish and non-Finnish replication cohorts.	174 Finnish preterm neonates, and 555 Finnish and 388 non-Finnish replication cohorts.	The risk of BPD was predicted by elevated plasma levels of CRP during the first week of life, and the SNP rs3093059 was modestly related to plasma CRP levels. SNP rs11265269 was found to be a risk factor for BPD, apart from other antenatal factors, suggesting a potential role for variants near CRP in BPD.
Yang et al., 2017 [18]	To assess the expression patterns of MMP and ARG in neonates with and without BPD.	Using the Gene-Cloud of the Biotechnology Information platform, a newborn gene expression dataset was re-analyzed from the GEO database.	299 samples.	Neonates with and without BPD showed upregulation of 28 and 11 ARGs, respectively. *MMP8, MMP9, MMP25, TIMP2*, and *TIMP3* had distinct expression patterns. MMP levels were associated with BPD severity. The up- and down-regulation of *THBS1, MMPs, TIMPs*, and *LEF1* induced BPD in preterm neonates by interfering with blood vessel development.
Torgerson et al., 2018 [19]	To identify variants, genes, and pathways associated with survival without BPD.	An ancestry GWAS study.	387 high-risk neonates treated with inhaled nitric oxide.	The most common individual variant found in all newborns was located in the intron of *NBL1* rs372271081. A variation in genes involved in immune/inflammatory processes in response to infection and mechanical ventilation was revealed, whereas examination of genes upregulated in BPD lungs revealed an association with variants in a cytokine linked to fibrosis and interstitial lung disease.
Wang et al., 2022 [20]	To investigate the RNA profiles in exosomes in umbilical cord blood of neonates with and without BPD.	The expression profiles of circRNAs, lncRNAs, mRNAs, and the proliferation of LPS-BEAS-2B, HUVECs, and cytokines in umbilical cord blood were examined using microarray analysis, co-expression networks, and Western blot analysis. To investigate exosomal circRNAs, lncRNAs, and mRNAs, GO enrichment and KEGG pathway analysis were performed.	4 neonates with and 4 without BPD.	317 circRNAs, 104 lncRNAs, and 135 mRNAs from umbilical-cord-blood-derived exosomes of neonates with versus without BPD demonstrated substantial differential expression. LPS significantly reduced BEAS-2B cells’ viability and increased HUVECs’ inflammatory responses. 455 circRNA/lncRNA–miRNA–mRNA interaction networks that could be associated with BPD were predicted. The findings demonstrated a strong correlation between the development of endothelium or epithelial cells and the GO keywords and KEGG pathways, which mostly involved differentially expressed exosomal RNAs.
Carrera et al., 2015 [21]	To identify non-common variants with a stronger phenotypic effect on BPD.	For exome sequencing, an Illumina HiSeq 2000 was employed. The ToppGene Suite was used to undertake a prioritization analysis revealing genes previously linked to BPD.	26 preterm unrelated neonates with BPD.	3369 new variants were found, with a median of 400 variations per sample. Several top-candidate genes were identified and confirmed with Sanger sequencing.
Wang et al., 2022 [22]	To determine early epigenetic indicators linked to BPD and investigate the association between gestational age, birth weight, and blood cell type in preterm neonates.	Cord blood DNA was added to Illumina 450 K methylation arrays. DNA methylation profiles were used to predict blood cell types. A multivariable regression analysis clarified the CpGs linked to the risk of BPD. Differently expressed genes were found by cDNA microarray analysis of cord blood RNA.	14 neonates with and 93 without BPD.	High-NRBC blood samples showed a hypomethylation profile, and the estimated NRBC % was favorably correlated with cg05575921 and negatively correlated with birth weight and gestational age. In cord blood, BPD-associated CpGs were enriched for hematopoiesis and lung maturation pathways. The incidence of stochastic epigenetic mutations at birth was considerably higher in neonates with BPD. Changes in the transcriptome of cord blood cells were indicative of the development of BPD.
Magagnotti et al., 2013 [23]	To explore protein changes in the BALF.	Data were analyzed using principal component analysis.	12 preterm neonates.	Deregulated proteins in preterm neonates and less abundant calcium-signaling-related proteins, consistent with BPD severity, were discovered.
Ahmed et al., 2022 [24]	To investigate the feasibility of non-invasively collected urine samples for proteomics from neonates at risk for BPD.	A high-throughput urine proteomics methodology, on urine collected within 72 h of birth.	21 neonates with BPD and 21 controls.	The urine proteomics method successfully detected several BPD-related alterations in the urine proteome that mirrored anticipated changes in the blood proteome, and several urine proteins predicted the risk of BPD.

circRNA, circular RNA; RDS, respiratory distress syndrome; GO, Gene Ontology; KEGG, Kyoto Encyclopedia of Genes and Genomes; miRNA, microRNA; GWAS, Genome-Wide Association Study; BPD, bronchopulmonary dysplasia; SNPs, single-nucleotide polymorphisms; CRP, C-reactive protein; MMP, matrix metalloproteinase; ARG, angiogenesis-related genes; GEO, Gene Expression Omnibus; lncRNAs, long noncoding RNAs; mRNAs, messenger RNAs; LPS, lipopolysaccharide; BEAS, bronchial epithelial cells; HUVECs, human umbilical vein endothelial cells; NRBC, nucleated red blood cells; EWAS, Epigenome-Wide Association Study; BALF, broncho-alveolar lavage fluid.

**Table 3:** Case-report studies in neonatal respiratory diseases.

Reference	Aim	Method	Population	Outcome
Cystic Fibrosis				
Esposito et al., 2024 [25]	To investigate if neonatal screening programs’ early discovery could enhance the prognosis for neonates with cystic fibrosis.	An Alu element insertion was found in exon 15 of the CFTR gene using bioinformatics tools and biological methods based on Sanger sequencing. This insertion was previously missed in routine next-generation sequencing analysis.	1 neonate.	The CFTR protein’s structure, splicing patterns, and gene expression are all significantly impacted by the Alu element insertion.

CFTR, cystic fibrosis transmembrane conductance regulator.

**Table 4:** Original studies in neonatal cardiovascular disorders.

Reference	Aim	Method	Population	Outcome
Congenital heart defects				
Bahado-Singh et al., 2015 [26]	To assess methylation of cytosines in CpG and to examine variations in genome-wide DNA methylation in neonates with CHDs in comparison to controls.	DNA from neonates with CHDs, such as hypoplastic left heart syndrome, ventricular septal defect, atrial septal defect, pulmonary stenosis, coarctation of the aorta, and Tetralogy of Fallot, as well as controls, were subjected to a genome-wide DNA methylation analysis.	60 neonates with CHD and 32 controls.	Several genes throughout the genome showed highly significant variations in cytosine methylation for each CHD. Anatomical structure morphogenesis was found in the overall GO analysis. For the diagnosis of CHD, methylation of individual cytosines in CpG demonstrated good diagnostic accuracy.
Bahado-Singh et al., 2020 [27]	To examine whether notable epigenetic modifications exist in isolated, non-syndromic CoA.	The Illumina HumanMethylation450 BeadChip arrays were used to analyze the genome-wide DNA methylation. To detect the molecular and disease pathways that were epigenetically dysregulated, ingenuity pathway analysis was used. Six artificial intelligence technologies were employed to detect CoA using methylation data.	24 isolated, non-syndromic CoA cases and 16 controls.	In CoA individuals, methylation alterations of significant magnitude were detected at 65 distinct CpG sites spread over 75 genes. Important cardiovascular developmental genes and biological processes, including aberrant cardiovascular system morphology, left ventricular dysfunction, heart conduction abnormality, thrombus formation, and coronary artery disease, were found to have epigenetic changes according to GO analysis.
Radhakrishna et al., 2018 [28]	To investigate the epigenetic alterations in neonates with TOF.	Using Illumina Infinium HumanMethylation450 BeadChips, a genome-wide methylation test was conducted.	24 non-syndromic TOF cases and 24 unaffected matched controls.	For 51 CpG sites, the difference in CpG methylation between TOF and controls was ≥10%, indicating biological importance. Significant biological processes, such as the formation of CHD, cardiomyopathy, diabetes, immunological response, inflammation, and other likely pathways in CHD development, were linked to these differentially methylated genes by GO analysis. In neonates with TOF, several genes, including *ABCB1*, *PPP2R5C*, *TLR1*, *SELL*, *SCN3A*, *CREM*, *RUNX*, and *LHX9*, were differently methylated.
Rashkin et al., 2022 [29]	To investigate the genetic architecture of OHDs.	Participants in the National Birth Defects Prevention analysis were the subjects of a genome-wide association analysis. Individual case-control studies on mothers and neonates and transmission/disequilibrium testing in whole case-parental trios were performed.	3978 neonates (2507 neonates as replication population).	The SLC44A2 SNP, rs2360743, was found to be substantially correlated with OHD. A SNP in CAPN11 (rs55877192) has been suggested to be linked to OHD. No SNPs were found to be genome-wide-significant in the case-control comparisons, and one SNP (rs188255766) in the neonatal study was associated with OHDs.
Mouat et al., 2023 [30]	To determine whether newborn dried blood spots from DS neonates with major CHDs differ in DNA methylation from DS neonates without CHDs.	To quantify DNA methylation, whole-genome bisulfite sequencing and the Illumina EPIC array were employed. DMRs between DS-CHD and DS non-CHD neonates were found through analysis of global CpG methylation. Genomic coordinate analysis and gene mapping were used to assess the GO enrichment of CHD DMRs. DMRs were also examined against methylation levels in DS versus usual development. Machine learning algorithms were utilized.	86 samples.	Compared to DS non-CHD males, DS-CHD males had global CpG hypomethylation. In DS versus non-DS samples, there was a higher proportion of CHD-associated DMRs with differential methylation.
Huang et al., 2023 [31]	To provide a case-mother and control-mother design for GATI-MFG.	Common and rare variations in a two-phase GWAS of CHDs were examined with GATI-MFG.	1306 control mother–infant pairs and 947 case mother–infant pairs with CHD.	Two genes on chromosome 17, *TMEM107* and *CTC1*, were found to have a significant correlation with CHD in common variants analysis for 23,035 genes. Heterotaxy was linked to the gene *TMEM107*. In the simulations, GATI-MFG performed better overall than the single-variant test, and the application to samples confirmed the link between *TMEM107* and *CTC1* and CHDs.
Wang et al., 2023 [32]	To investigate the distribution of hereditary factors in neonates with CHD.	To determine the incidence of genetic diagnoses and the corresponding neonatal outcomes, next-generation sequencing findings and medical records were retrospectively reviewed in CHD cases from the China Neonatal Genomes Project (2016–2021).	1795 neonates.	Genetic diagnoses were made for 269 of the neonates, including 172 copy number variations and 97 pathogenic mutations, which could account for the phenotype of CHDs. Compared to clinical exome sequencing, trio-whole-exome sequencing had a greater detection rate. A subsequent study revealed that the genetic diagnosis rate in the group who died was higher than in the group that survived.

CHD, congenital heart defects; GO, Gene Ontology; CoA, coarctation of aorta; TOF, Tetralogy of Fallot; OHD, obstructive heart disease; DS, down syndrome; SNP, single-nucleotide polymorphism; DMR, differentially methylated regions; GATI-MFG, gene-based association test of interactions for maternal-fetal genotypes; GWAS, Genome-Wide Association Study.

**Table 5:** Original studies in neonatal gastrointestinal disorders.

Reference	Aim	Method	Population	Outcome
Gut microbiota				
Wang et al., 2016 [33]	To investigate the expression of lactation-related miRNAs in microvesicles isolated from human umbilical cord blood.	Western blotting, transmission electron microscopy, and nanoparticle tracking analysis. Bioinformatics techniques for GO, miRNA target prediction, signaling pathway analysis, and lactation-related miRNAs.	70 participants.	After profiling 337 miRNAs in human umbilical cord blood microvesicles, bioinformatics analysis revealed that 85 of them were connected to lactation. HBL-100 cells absorbed microvesicles after 4 h in culture, and 96 h later, microvesicle-exposed cells showed much higher β-casein secretion. The study indicated that umbilical cord blood microvesicles may play a significant role in fetal–maternal interactions by mediating β-casein secretion through miRNAs.
Parnanen et al., 2022 [34]	To investigate how early formula intake affects newborns’ ARG burden.	A generalized linear model was constructed using cross-sectionally sampled neonatal gut metagenomes to examine the effect of food on ARG loading. Neonatal metagenomes collected from public databases were used to cross-validate the model.	46 neonates.	When compared to neonates that were exclusively fed human milk, the formula-receiving group had a 69% greater relative abundance of ARGs carried by gut bacteria. Additionally, the formula-fed neonates had much fewer common bacteria—such as Bifidobacteria, which may be beneficial to health—than other neonates.
Liu et al., 2023 [35]	To examine the metabolite levels and gut microbiota dysbiosis characteristics in neonates with WMI.	LC-MS/MS, diffusion tension imaging, and 16S rRNA gene sequencing were used as part of a multi-omics approach to finding quantifiable and useful biomarkers for WMI. The Illumina MiSeq PE300 platform was utilized to perform paired-end sequencing on a pooled set of purified amplicons. The Majorbio Cloud platform was utilized for the bioinformatic investigation of the gut microbiota.	23 neonates with and 48 without WMI.	Between WMI and non-WMI neonates, there was a significant and differential expression of 139 metabolic markers. The WMI group exhibited significant downregulation of 17 metabolic pathways, including the production of arginine and main bile acids, according to KEGG pathway enrichment analysis. By downregulating metabolites, such as cholic acid, allocholic acid, and 1,3-butadiene, *Staphylococcus* species may have an impact on WMI. *Acinetobacter* and *Bacteroidetes* in the gut microbiota may structurally change white matter by upregulating compounds, such as cinobufagin.
Letourneau et al., 2024 [36]	To explore the association between microbiome composition and biomarkers and the risk of developing specific diseases.	pH, redox, SCFA content, and microbiome composition were analyzed by 16S rRNA gene amplicon sequencing. These outcomes were compared to the clinical data.	11 neonates.	Neonates born earlier or exposed to antibiotics exhibited increased fecal pH and increased redox. The variations in SCFA content, which was associated with pH and age, could be the cause of these discrepancies. Gestational age, pH, redox, and birth weight were also linked to the composition of the microbiome.
Necrotizing enterocolitis				
Liu et al., 2022 [37]	To detect differentially expressed genes in neonates with NEC.	The GEO2R database, GO, and KEGG were used to identify differentially expressed genes. DAVID and STRING were used to examine pathway enrichment and protein interactions. Cytoscape software was used to identify the important protein interaction modules and core network genes.	9 neonates.	The findings showed that the differentially expressed genes that were upregulated were associated with protein dimerization activity, whereas the genes that were downregulated were associated with cholesterol transporter activity. The differentially expressed genes were substantially concentrated in pathways related to fat digestion and absorption and metabolism, according to KEGG enrichment analysis. In the protein network interaction map, nine important genes were found using STRING analysis: *EPCAM*, *CDH1*, *CFTR*, *IL-6*, *APOB*, *APOC3*, *APOA4*, *SLC2A*, and *NR1H4*.
Chen et al., 2021 [38]	To investigate the effects of human-milk-derived exosomes and elucidate their lipid expression profiles.	Isolated and quantified exosomes from healthy mothers’ milk.	6 participants.	When term and preterm milk exosomes were administered, the severity of NEC in vivo was lessened, and epithelial proliferation and migration were greatly increased in vitro. Exosomes generated from human milk, both preterm and term, contained 395 different types of lipids. The function of intestinal epithelial cells was regulated by the top 50 lipids through the extracellular signal-regulated kinase/mitogen-activated protein kinase (ERK/MAPK) pathway.
Zhang et al., 2021 [39]	To explore the mechanism of the pathogenesis of NEC.	Gene expression levels were quantified using RNA sequencing. The genes that were differentially expressed were found using the DESeq2 program. Gene set enrichment analysis was used to characterize the biological function, pathways, transcription factors, and immune cells that were dysregulated in NEC.	9 neonates with NEC and 5 controls.	It was discovered that immune-associated pathways were substantially activated in NEC, although several pathways connected to cellular responses to external stimuli were inactivated. NEC showed a high activation of transcription factor genes associated with inflammation, including STAT1, STAT2, and IRF2. The diversity of immune cells in NEC suggested that an inflammatory response related to NEC could be triggered by both innate and adaptive immune systems.
Tremblay et al., 2021 [40]	To examine RNA-Seq data from the analysis of intestinal specimens of preterm neonates diagnosed with NEC.	Previous data were analyzed using function enrichments with GO and KEGG to find biological and functional processes that were involved in the pathophysiology of NEC.	9 neonates with NEC and 5 controls.	The most important biological pathways that were over-represented in NEC neonates, according to gene set enrichment analysis, were strongly associated with the innate immune processes. The expression of inflammatory genes associated with the IL-17 pathway was reduced in the colon of neonates with NEC. These genes included pro-inflammatory cytokines, chemokines, and antimicrobials.
Hirschsprung’s disease				
Niu et al., 2019 [41]	To benefit from RNA-Seq information obtained from the examination of intestinal tissues from premature neonates with Hirschsprung’s disease.	The public dataset’s DEGs were examined. Database for Annotation, Visualization, and Integrated Discovery was used to carry out GO and KEGG pathway enrichment analyses. Weighted gene co-expression network analysis was used to create the co-expression network between lncRNAs and mRNAs.	8 neonates with Hirschsprung’s disease and 8 controls.	11 KEGG pathways, including protein digestion and absorption and mineral absorption, were identified among the 864 DEGs enriched in 19 GO biological activities. A network including 8 modules and 177 genes was created to co-express lncRNAs and mRNAs. Hub lncRNAs were found, and they included DRAIC, LINC00619, LINC00924, and LINC00261. Hub mRNAs were primarily concentrated in the calcium signaling route, p53 signaling pathway, cancer pathways, and BDKRB, ITGA6, and TNNC1 pathways. An additional independent set of GSE96854 data satisfactorily validated the hub mRNA expressions.
Feng et al., 2024 [42]	To investigate how the ZEB2/Notch-1/Jagged-2 pathway and ENPCs function in the development of HAECs.	Lipopolysaccharide was used to induce ENPCs. ZEB2/Notch-1/Jagged-2 expressions were quantified by Western blot and RT-qPCR. The differentiation and proliferation of ENPCs were evaluated using immunofluorescence and cell-counting kit-8 tests. Bioinformatics analysis and co-immunoprecipitation were utilized to investigate the ZEB2 and Notch-1 interaction.	8 neonates.	HAEC colon tissues had higher levels of lipopolysaccharide, along with downregulated ZEB2 and elevated Notch-1/Jagged-2 expression. In ENPCs, lipopolysaccharide treatment caused proliferation and differentiation abnormalities, increased Notch-1/Jagged-2 expression, and downregulated ZEB2 expression. In lipopolysaccharide-induced ENPCs, overexpression of ZEB2 exacerbated inflammation and dysfunction while suppressing Notch-1/Jagged-2 signaling. Overexpression of Notch-1 exacerbated lipopolysaccharide-induced dysfunction; thus, playing a role in HAEC

GO, Gene Ontology; ARG, antibiotic-resistance genes; WΜΙ, white matter injury; KEGG, Kyoto Encyclopedia of Genes and Genomes; GO, Gene Ontology; lncRNAs; long noncoding RNAs; mRNAs, messenger RNAs; SCFA, short-chain fatty acids; NEC, necrotizing enterocolitis; GEO2R, Gene Expression Omnibus; DEGs, differently expressed genes; ENPCs, enteric neural precursor cells; HAEC, Hirschsprung’s-associated enterocolitis.

**Table 6:** Original studies in neonatal sepsis.

Reference	Aim	Method	Population	Outcome
Mustarim et al., 2019 [43]	To determine the association between gene polymorphism of *BPI* rs4358188, *CD14* rs2569190, *IL1β* rs1143643, or *MMP16* rs2664349 and neonatal sepsis.	Genomic DNA samples obtained from neonates, continued with PCR examination, sequencing, and bioinformatics analysis.	30 neonates with and 30 neonates without sepsis.	A statistically significant correlation was observed between the *IL1β* rs1143643 G>A gene polymorphism and the frequency of newborn sepsis.
Bu et al., 2020 [44]	To identify upregulated and downregulated mRNAs, and lncRNAs in neonatal sepsis.	PPI networks to identify key regulators in neonatal sepsis, including *ITGAM*, *ITGAX*, *TLR4*, *ITGB2*, *SRC*, *ELANE*, *RPLP0*, *RPS28*, *RPL26*, and *RPL27*.	68 neonates.	Neonatal sepsis was shown to have 1128 upregulated mRNAs, 1008 downregulated mRNAs, 28 upregulated lncRNAs, and 61 downregulated lncRNAs. Neonatal sepsis progression was found to be significantly influenced by *HS.294603*, *LOC391811*, *C12ORF47*, *LOC729021*, *HS.546375*, *HNRPA1L-2*, *LOC158345*, and *HS.495041*, according to lncRNA co-expression analysis. The regulation of cellular extravasation, acute inflammatory response, ribosome, RNA transport, spliceosome, and macrophage activation of NF-kappa B, TNF, HIF-1, and Toll-like receptor signaling pathway was demonstrated by bioinformatics analysis to involve DEGs.
Navarrete et al., 2021 [45]	To identify DNA methylation traits in neonatal sepsis.	Two different bioinformatic methods were used to find DMRs using data from Methylation EPIC 850K BeadChip arrays: mCSEA (supervised) and DMRcate (unsupervised).	15 neonates.	Neonates with sepsis showed variations in their methylation levels. Disparities were noted between both early- and late-onset sepsis.
Yan et al., 2022 [46]	To identify the optimal biomarkers in the progression of neonatal sepsis.	GSVA for biological function and pathway alterations between neonates with and without sepsis. To measure variations in immune cell infiltration between the two groups, CIBERSORT, ROC analysis, and the LASSO model were used.	22 neonates with and 16 neonates without sepsis.	Sepsis samples were examined for 85 upregulated and 40 downregulated overlapping DEGs. According to the GSVA data, DEGs mostly influenced the upregulation of metabolism-related activities and inflammation, as well as the suppression of adaptive immune responses in sepsis. 57 genes were chosen and included in a LASSO model with an AUC > 0.9 in both discovery sets. RT-qPCR was utilized to extract and validate five genes that were identified as having the best diagnostic performance among the gene signatures in clinical samples. Three genes were shown to be important biomarkers for sepsis, *SLC2A3*, *OSCAR*, and *CD3G*.
Ciesielski et al., 2022 [47]	To identify late-onset sepsis-associated genetic variants.	A GWAS investigation was carried out. In the entire sample and sex-stratified studies, a connection with both autosomal and X-chromosome variations was examined.	224 late-onset sepsis cases and 273 controls.	In at least one investigation, 71 SNPs were linked to neonatal sepsis. Variants from single-sex analysis were not linked to sepsis in the other sex, whereas stratified analyses by gender showed relationships with several SNPs. Notch signaling is over-represented among the genes connected to these SNPS, according to pathway studies.
Liu et al., 2024 [48]	To investigate the characteristics of intestinal metabolomics and non-invasive biomarkers for late-onset sepsis.	Stool samples from septic and healthy preterm neonates were analyzed by liquid chromatography–mass spectrometry.	60 neonates.	Thirteen pathways accounted for the majority of the 123 distinct metabolites. N-methyldopamine, cellulose, glycine, N-ribosylnicotinamide, Gamma-glutamyltryptophan, and 1-alpha, 25-dihydroxycholecalciferol significantly changed and demonstrated distinct diagnostic values as non-invasive biomarkers for late-onset sepsis.
Das et al., 2024 [49]	To investigate the blood profile of very preterm neonates across episodes of sepsis.	Multi-parameter flow cytometry, single-cell RNA sequencing, and plasma analysis.	19 neonates.	Sepsis was characterized by a constantly shifting blood immunological signature, which included lower dendritic cell frequencies, myeloid cell HLA-DR expression, and lymphopenia. Even in cases where CRP was normal, single-cell RNA sequencing revealed elevation of amphiregulin in leukocyte populations during sepsis, which was associated with clinical indications of sepsis.
Hang et al., 2024 [50]	To investigate whether PCD can be used as a biomarker for neonatal sepsis.	The DEGs for neonatal sepsis and controls, as well as PCD-related genes from 12 distinct patterns, were obtained by using the GEO public repository. Three sophisticated machine learning methods were used: RF, SVM-RFE, and LASSO. PPI networks, artificial neural networks, and consensus clustering were utilized to validate the results.	26 neonates with sepsis and 37 controls.	A total of 49 genes showed a junction between the genes linked to PCD and the DEGs. It was shown that six genes were shared by PCD-associated genes and DEGs. After merging differential expression profiles, a diagnostic model was created, and consensus clustering and artificial neural networks were used to validate it. Notable differences were found in the immune infiltration study of patients with neonatal sepsis. The ceRNA network demonstrated a complex regulatory interaction based on the marker genes that were discovered.
Zhao et al., 2024 [51]	To examine the expression patterns of certain miRNAs and assess their diagnostic usefulness for sepsis early identification and management.	PPI studies and GO enrichment were conducted with the assistance of differentially expressed miRNAs.	43 neonates with sepsis and 59 controls.	Three miRNAs (mir-223-3p, mir-15a-5p, and mir-17-5p) were significantly downregulated in serum, while mir-146a-5p, mir-1-3p, and mir-16-5p were elevated in neonates with early-onset sepsis. These miRNAs had a moderate diagnostic value, and the diagnostic panel made up of miR-15a-5p, miR-223-3p, and miR-16-5p had a significantly higher diagnostic value, suggesting that the combination of these miRNAs may be a useful biomarker for the clinical diagnosis of early-onset sepsis. Based on GO enrichment analysis, the majority of target gene-encoded proteins were found in the cytosol as regulators in protein binding.

BPI, bactericidal permeability increasing; CD, cluster of differentiation, IL, interleukin, MMP, matrix metalloproteinase; lncRNAs, long noncoding RNAs; PPI, protein–protein interaction; DEGs, differentially expressed genes; GSVA, gene set variation analysis; GWAS, Genome-Wide Association Study; SNP, single-nucleotide polymorphisms; ROC, receiver operating characteristic; AUC, area under the curve; CRP, C-reactive protein; PCD, programmed cell death; RF, radiofrequency; SVM-RFE, support vector machine-recursive feature elimination; ceRNA, competing endogenous RNA; GO, Gene Ontology; miRNAs, microRNAs.

**Table 7:** Original studies in neonatal neurology.

Reference	Aim	Method	Population	Outcome
Hypoxic-Ischemic Encephalopathy				
Chu et al., 2006 [52]	To investigate the metabolomic patterns of newborn urine samples with clinical indications of severe hypoxia at birth.	Bioinformatic techniques were used to analyze the urinary metabolite profiles, which were determined using high-throughput mass spectrometry. Hierarchical clustering analysis was used to identify the metabolomic discriminators between good and poor newborn outcomes.	256 neonates.	Inhibited biochemical networks involved in macromolecular production were correlated with HIE. Elevated levels of eight urine organic acids in different biochemical pathways were found to be highly sensitive and specific indicators of the prognosis of neurodevelopmental impairment: ethylmalonate, 3-hydroxy-3-methylglutarate, 2-hydroxy-glutarate, and 2-oxo-glutarate were linked to favorable neonatal outcomes, while glutarate, methylmalonate, 3-hydroxy-butyrate, and orotate were associated with unfavorable outcomes.
Zhu et al., 2020 [53]	To identify potential biomarkers of neonatal HIE.	The iTRAQ method was used. The identified differentially expressed proteins were subjected to bioinformatics investigations, such as GO and KEGG pathway enrichment analysis, to assess their potential traits and capabilities.	12 neonates with HIE and 4 controls.	When mild, moderate, and severe HIE were compared to healthy controls, 51 frequently differently expressed proteins were found. The two most markedly upregulated markers in HIE patients were haptoglobin and S100A8, which were further confirmed by Western blotting and real-time PCR. The proteins that were differently expressed were abundant in complement and coagulation cascades and represented a variety of biological processes, cellular components, and molecular activities.
Friedes et al., 2023 [54]	To investigate mechanisms of injury and recovery in neonatal encephalopathy.	LC/MS/MS was used to undertake targeted metabolomic analysis of plasma. Using a 193-plex targeted metabolite test that covers over 366 metabolic pathways, plasma samples underwent LC/MS/MS metabolomic profiling. The two-year neurodevelopmental outcomes assessed by the Bayley Scales of Infant and Toddler Development III were compared to metabolite levels.	30 healthy term neonates and 45 neonates with neonatal encephalopathy.	57 of the 193 metabolites had analysis results that satisfied the predetermined quality control standards. Aminoacyl-tRNA biosynthesis, arginine production, and the metabolism of various amino acids were important KEGG pathways. Regression models showed a significant relationship between betaine and the cognitive and motor Bayley-III composite scores and between histidine and C6 sugar amine and the linguistic, motor, and cognitive domains. The Bayley-III cognitive, motor, and language scores showed a significant improvement in model performance when histidine, C6 sugar amine, and betaine were included in a clinical regression model based on the Sarnat score.
Inborn Errors of Metabolism				
Tangeraas et al., 2020 [3]	To evaluate the performance of the Norwegian expanded NBS, including a total of 25 conditions (21 IEMs).	Second-tier biochemical testing, genetic confirmation utilizing DNA isolated from the original dried blood spots, and the use of the region 4 Stork post-analytical interpretive tool (R4S)/Collaborative Laboratory Integrated Reports 2.0 for screening for 21 IEMs.	461,369 neonates.	When the NBS findings were obtained, 21% of the true-positive patients had symptoms; nonetheless, in two-thirds of these cases, the screening result guided the precise diagnosis. There was an increase in the annual positive predictive value from 26% to 54%. After expanded NBS was implemented, the incidence of IEMs increased by 46%, mostly as a result of the discovery of attenuated phenotypes.
Hagemeijer et al., 2023 [55]	To improve the detection of LSDs (oligosaccharidoses, sphingolipidoses, and mucolipidoses).	An open-source, iterative bioinformatics method, UHPLC/HRAM mass spectrometry screening technology.	94 neonates.	Numerous LSDs were identified, including sialidosis, galactosialidosis, fucosidosis, α-mannosidosis, β-mannosidosis, α-N-acetylgalactosaminidase deficiency, GM1 gangliosidosis, GM2 gangliosidosis, and mucolipidosis II/III. Other disorders, such as sialic acid storage disease, MPS type IV B, NGLY1 congenital disorder of deglycosylation, and GSD II (Pompe disease), were associated with abnormal urine oligosaccharide excretions. In addition to glucose tetrasaccharide (Glc4), heptahexose (Hex7) was found to be a potential urine biomarker for the latter disease, which could be used for the diagnosis and follow-up of young-onset Pompe disease cases.
Sabi et al., 2024 [56]	To find further biomarkers to distinguish healthy newborns from falsely suspected GA-1.	Through the NBS program, samples from matched controls and falsely suspected GA-1 neonates were gathered. Utilizing LC-HRMS, untargeted metabolomics was carried out to provide biomarker and pathway analyses for altered metabolites.	47 neonates.	In transient GA-1, there were 582 up- and 546 down-regulated metabolites, while 155 endogenous metabolites showed notable differences when compared to the control group. Novel metabolic biomarkers that were altered in conjunction with the transient elevated C5DC levels were revealed. These biomarkers included N-palmitoylcysteine, heptacarboxyporphyrin, 3-hydroxylinoleoylcarnitine, and monoacylglyceride, and perturbed metabolic pathways, such as thiamine and sphingolipid metabolism.
Cognitive Outcome				
Chung et al., 2024 [57]	To examine 4 prediction models for cognitive or motor function at 24 months of corrected age separately at each follow-up visit, 2 for the 6-month and 2 for the 12-month visits.	Regression and delay models were employed at 6 and 12 months of age, respectively. Models were created using an evolutionary-derived machine learning technique, called EL-NDI, and compared to models constructed using random forest, SVM, and LASSO regression.	1244 very preterm infants in the developmental set and 763 and 1347 in the two validation cohorts, respectively.	While other models required 29 or more variables to attain comparable performance, EL-NDI used 4–10 variables. The AUC of the EL-NDI for models at six months of follow-up was 0.79–0.83 for motor regress with four factors and 0.76–0.81 for cognitive regress with four variables. In models with ten variables for cognitive delay and four variables for motor delay at a year and a half, respectively, the AUC of EL-NDI was 0.75–0.78 and 0.73–0.82.

HIE, hypoxic-ischemic encephalopathy; iTRAQ, isobaric tags for absolute and relative quantification; GO, Gene Ontology; KEGG, Kyoto Encyclopedia of Genes and Genomes; LC/MS/MS, liquid chromatography with tandem mass spectrometry; NBS, newborn screening program; IEMs, inborn errors of metabolism; LSDs, lysosomal storage disorders; UHPLC/HRAM, ultra-high-performance liquid chromatography/high-resolution accurate mass; GA-1, glutaric aciduria type-1; LC-HRMS, liquid chromatography–high-resolution mass spectrometry; SVM, support vector machines; AUC, area under the curve.

**Table 8:** Case-report studies in neonatal neurology.

Reference	Aim	Method	Population	Outcome
Inborn Errors of Metabolism				
Maryami et al., 2023 [58]	To evaluate the genetic cause of the metabolic crisis and death in a 3-day-old neonate,	WES and Tandem MS.	1 neonate.	A homozygous missense variation (NM_000060.4(BTD):c.1330G > C) in exon 4 of the BTD gene was identified by WES as the cause of a partial biotinidase deficiency. A homozygous significant deletion in the *PCCA* gene was detected with the IGV program, spanned from PCCA’s intron 11 to 21, and caused a premature termination codon as well as the activation of nonsense-mediated mRNA decay. The deletion of the active site and important functional regions of the protein was shown by homology modeling of the mutant *PCCA* gene causing severe acute early-onset form of propionic acidemia.
Maryami et al., 2023 [59]	To confirm pathogenicity in a two-day-old neonate presenting early-onset metabolic crisis and death.	Sanger sequencing, linkage analysis, WES, and in silico assessment of the impact of the variations on the structure and function of proteins.	1 neonate.	On chromosome 12, in exon 7, NM_052845.4 (MMAB):c.557G > A, p.Arg186Gln, was found to have a homozygous missense variant. A thorough bioinformatics investigation revealed modifications in protein–ligand and protein–protein interactions, as well as a notable decrease in variant stability. In contrast to the known pathogenic variant, c.556C > T, p.Arg186Trp, the variant c.557G > A, p.Arg186Gln showed more changes in the secondary structure and less binding to the ATP and B12 ligands.
Maryami et al., 2023 [60]	To assess the acute, early-onset metabolic disruption and mortality in two unrelated neonates.	WES analysis, Sanger sequencing, homology modeling, and in silico bioinformatics analysis were employed to assess the effects of variants on protein structure and function.	2 neonates.	In the pyruvate carboxylase gene of each neonate, WES identified two unique homozygous variants, p.G303Afs*40 and p.R156P. The p.R156P was classified as a VUS and the p.G303Afs*40 as probably pathogenic. The wild-type protein and the p.R156P and p.R156Q variants differed from each other according to protein secondary structure prediction. R156P and R156Q were both predicted to be harmful variations by investigation of SNP impacts on protein utilizing Polyphen-2, SNAP2, FATHMM, SNPs, and GO servers.
Forte et al., 2023 [61]	To report on a compound heterozygote in the GALT gene for a novel missense variant (p.A303D) and a known pathogenic variant (p.K285N).	Segregation analysis and bioinformatics analysis to predict the impact of the missense variant on the structure and stability of the GALT protein.	A two-week-old female neonate.	The neonate inherited the p.K285N pathogenic variant from her father and the p.A303D variant from her mother. A bioinformatics analysis to predict the impact of the p.A303D missense variant on the structure and stability of the GALT protein revealed that it may be pathogenic.
Wei et al., 2024 [62]	To examine the genetic cause of familial autosomal dominant PKD1.	The proband’s PKD1 underwent WES analysis, Sanger sequencing, and bioinformatics analysis. The gene’s conservation and the pathogenicity of the mutations were assessed using SIFT, Polyphen2, and MutationTaster. The protein structures of PKD1 and mutant neonate proteins were predicted and mapped using SWISS-MODEL.	An affected Chinese family.	WES of the proband revealed a new c.9484delC (p.Arg3162Alafs*154) mutation of the *PKD1* gene, which was validated by Sanger sequencing of his sister. The members of the healthy pedigree did not exhibit the same mutation. The c.9484delC mutation was not found in 100 patients with normal and end-stage renal disease who were randomly screened. According to the bioinformatic study, the mutation resulted in a shift in the termination codon and the 3162nd amino acid substitution of alanine for arginine. The modified amino acids were shown to be significantly conserved in mammals by cluster analysis.

WES, whole-exome sequencing; VUS, variation of unknown significance; SNP, single-nucleotide polymorphism; MS, mass spectrometry; IGV, Integrative Genomics Viewer; GO, Gene Ontology; GALT, galactose-1-phosphate uridylyltransferase, PKD-1, polycystic kidney disease-1.

**Table 9:** Original studies of miscellaneous applications of bioinformatics in neonatal medicine.

Reference	Aim	Method	Population	Outcome
Spina bifida				
Lu et al., 2006 [63]	To detect SNPs of PAX3 potentially associated with syndromic neural tube defects.	Both the upstream genomic area and the conserved paired-box domain of PAX3 underwent resequencing, and the SNPs that were found were assessed as haplotypes. Further research was performed on the relationships between spina bifida risks and haplotypes for particular gene areas.	74 infants with spina bifida (cases) and 87 nonmalformed neonatal controls.	Nineteen SNPs were found. The National Centre for Biotechnology Information database has allele frequencies for fifteen of the SNPs found in controls. It was discovered that among Hispanic Whites that the PAX3 gene variant T-1186C (rs16863657) and its corresponding haplotype, TCTCCGCCC of nine SNPs, were linked to an elevated incidence of spina bifida.
Hypoxia-Induced Acute Kidney Injury				
Pan et al., 2023 [64]	To investigate the expression of new noncoding circRNAs between hypoxia-induced AKI neonates and controls.	CircRNAs were analyzed using high-throughput sequencing. The role of circRNAs with differential expression was predicted using a bioinformatics study. qPCR was used to filter and identify the circRNAs that were differently expressed.	36 neonates.	A total of 296 circRNAs with differential expression were found. Of these, 112 circRNAs were considerably downregulated in the AKI group while 184 were noticeably elevated. For qPCR confirmation, the top five upregulated and downregulated circRNAs with the largest fold changes were chosen. When comparing the asphyxia-induced AKI group to the control group, hsa_circ_0008898 and hsa_circ_0005519 were considerably upregulated, while hsa_circ_0132279, hsa_circ_0112327, and hsa_circ_0017647 were significantly downregulated.
Retinopathy of Prematurity				
Shipton et al., 2024 [65]	To determine whether it is feasible to obtain and examine tear proteins from preterm children who are at risk of ROP, as well as any potential prognostic markers or tear proteins linked to the pathophysiology of ROP.	Tear samples from premature infants scheduled for ROP screening were collected using Schirmer’s technique. Mass spectrometry was used for proteomic analysis.	12 neonates.	Out of the 701 proteins that were discovered, 261 proteins were used for analysis. An increase in lactate dehydrogenase B chain in tears was linked to an increased risk of ROP. Two pairs of twins in the group had remarkably similar proteomes, and older neonates had higher concentrations of immunoglobulin complexes in their tear samples, which supported the validity of the analysis.
Rapid Trio Genome Sequencing				
Marom et al., 2024 [66]	To assess rtGS’s clinical value, diagnostic effectiveness, and viability in all of Israel’s neonatal intensive care units.	Sequencing analysis, reporting pathogenic, likely pathogenic, and highly suspected VUS. Questionnaires were used to evaluate the clinical utility.	130 neonates.	For disease-causing variations, the diagnostic effectiveness was 50%, for VUS suspected of being the cause, it was 11%, and for one unique gene candidate, it was 1%. 12 chromosomal diseases, 52 monogenic disorders, and one newborn with uniparental disomy were among the mutations that caused the disease. 82% of respondents completed the clinical utility questionnaires overall. Of the respondents, 24 newborns (22%) had their medical care altered as a result of genomic testing.
Immunodeficiencies				
Pavey et al., 2024 [67]	To assess if genomic sequencing can improve the present newborn immunodeficiency screening program.	Clinician data and whole-genome sequencing were analyzed using phenotype-first and genotype-first methodologies. Using a phenotype-first strategy, neonates with clinical characteristics suggestive of immunodeficiency were found using electronic health data.	1349 newborn–parent trios.	13,476 unique variants, and 8502 unique projected variants affecting proteins were found. Five of the participants carried mutations that could be harmful and needed to be clinically correlated by an expert. A complement component 9 deficit was discovered genomically in one clinically asymptomatic person. Two of the symptomatic youngsters were found to have additional molecular diagnoses, and one was molecularly determined to have an immunodeficiency disease.

SNP, single-nucleotide polymorphism; CirRNSs, circular RNAs; AKI, acute kidney injury; ROP, Retinopathy of Prematurity; rtGS’s, rapid trio genome sequencing; VUS, variants of unknown significance.

**Table 10:** Case-report studies of miscellaneous applications of bioinformatics in neonatal medicine.

Reference	Aim	Method	Population	Outcome
Thyroid Disorders				
Baquedano et al., 2010 [68]	To describe the molecular effects of two new missense and splice-junction mutations discovered in the TSH-beta subunit gene in two patients suffering from anemia refractory to standard treatment and congenital central hypothyroidism.	The silent mutation suspected of causing aberrant RNA processing of the TSH-subunit gene was evaluated with the software NN Splice version 0.9. The pathogenicity of the missense variant C88Y was predicted using the sequence homology-based SIFT version 2.0.6 and the structure-based PolyPhen.	Two neonates.	A homozygous G to A nucleotide substitution was present in patient 1 at the 5’ donor splice location of exon/intron 2. As a result, codon 34 of the mature protein underwent a silent alteration. Sequence analysis for the previously described 313delT (C105Vfs114X) mutation and a second unique mutation in exon 3 that replaces A at cDNA nucleotide position 323 with G, resulting in a C88Y alteration, were both found to be compound heterozygotes in patient 2. PolyPhen and SIFT analysis, two distinct bioinformatics techniques, forecasted that C88Y would be a harmful substitution.
Congenital Dyserythropoietic Anemia Type II				
Zheng et al., 2024 [69]	To establish the diagnosis of CDA II.	WES.	A 1-month-old infant girl.	CDA II was diagnosed after WES detected a unique compound heterozygosity in the SEC23B gene. Several bioinformatics tools’ analyses indicated that the mutated proteins would be hazardous.
Progressive Familial Intrahepatic Cholestasis				
Khabou et al., 2024 [70]	To establish the diagnosis of PFIC.	Panel-target sequencing followed by exhaustive bioinformatics and modeling investigations.	6 unrelated Tunisian infants.	A novel p.Ala98Cys variant in the ATP-binding cassette subfamily G member 5 (*ABCG5*) gene, a first homozygous description of the p.Gln312His in the ABCB11 gene, and five known disease-causing variants. Based on their functionality and pathogenicity, two additional possible modifier variations in cholestasis-associated genes were kept. Five patients were diagnosed with PFIC2, and one patient had an unexpected sisterolemia diagnosis according to molecular results.

TSH, thyroid-stimulating hormone; CDA-II, congenital dyserythropoietic anemia type II; WES, whole-exome sequencing; PFIC, progressive familial intrahepatic cholestasis.

## Data Availability

Not applicable.
